# Prevalence and Severity of Depression and Its Association with Substance Use in Jimma Town, Southwest Ethiopia

**DOI:** 10.1155/2016/3460462

**Published:** 2016-03-16

**Authors:** Andualem Mossie, Dagmawi Kindu, Alemayehu Negash

**Affiliations:** ^1^Department of Biomedical Sciences, College of Health Sciences, Jimma University, Ethiopia; ^2^College of Health Sciences, Debre Tabor University, Ethiopia; ^3^Department of Psychiatry, College of Health Sciences, Jimma University, Ethiopia

## Abstract

*Background*. Depression is a significant contributor to the global burden of disease and affects 350 million people worldwide. Substance use could be the risk factor for depression.* Objective*. We aim to determine the prevalence and severity of depression and its association with substance use.* Methods*. A cross-sectional study was conducted on a sample of 650 respondents in Jimma town in March 2014. A multistage stratified sampling method was conducted. Structured questionnaire and Beck's Depression Inventory (BDI-II) scale were used for data collection. Data analysis was done using the SPSS Version 20.0 for Windows.* Results*. The participation rate of respondents was 590/650 (90.77%). The proportion of females was 300 (50.9%). The current prevalence of depression was 171 (29.0%). Based on the BDI-II grading of the severity of depression, 102 (59.6%) had mild, 56 (32.7%) had moderate, 13 (7.6%) had severe depression. In the present study, age of 55 years and above [OR = 5.94, CI: 2.26–15.58], being widowed [OR = 5.18, CI: 1.18–22.76], illiterates [OR = 9.06, CI: 2.96–27.75], khat chewing [OR = 10.07, CI: 5.57–18.25], cigarette smoking [OR = 3.15, CI: 1.51–6.58], and shisha usage [OR = 3.04, CI: 1.01–9.19] were significantly and independently associated with depression.* Conclusion*. The finding depicted that depression was a moderate public health problem. Advanced age, being widowed, illiterate, khat chewing, and cigarette and shisha smocking could be the potential risk factors for depression. Risk reduction is recommended.

## 1. Introduction

World Health Organization (WHO) has reported that about 450 million people worldwide suffer from mental illness and one in four people meets criteria of mental illness at some point in their life [1, 2]. Among the mental disorders, depression is a disease of the global burden affecting 350 million people worldwide [3]. A study conducted on adult populations of USA reported that lifetime prevalence of major depressive disorder was 16.2% and 12-month prevalence was 6.6% [4]. Surveys conducted in 16 European countries found that 27% of adult Europeans are affected by depression: at least one mental disorder in a 12-month period [5, 6]. The burden of the disease is higher by 50% in females than in males [7]. The prevalence of depression in low and middle income countries (LMIC) ranged from 11.1 to 53%. The burden is highest in Afghanistan, Middle East, and North and Sub-Saharan African countries [8]. It was reported that the prevalence of depression in Ethiopia is 9.1% [9].

Depression disorder presents with depressed mood, loss of interest or pleasure, decreased energy, feelings of guilt or low self-worth, disturbed sleep or appetite, poor concentration, problem of thinking and making decisions, and, in severe stages, recurring thoughts of death or suicide [10]. Depending on the number and severity of symptoms, a depressive episode can be categorized as mild, moderate, or severe. An individual with a mild depressive episode will have some difficulties in continuing with ordinary work and social activities but will probably not cease to function completely. On the other hand, it is very unlikely that the individual with severe depressive episode will be able to continue with social, work, or domestic activities, except to a very limited extent. As for moderate depression, the individual would normally have more than the five symptoms that are needed to make the diagnosis of depression. Moderate episodes have a severity that is intermediate between mild and severe depressions [11].

Depression has many possible causes, including mood disturbance, genetic vulnerability, chronic stressful life, use of psychoactive substances, and medical problems. It is believed that several of these forces interact to bring depression [10, 11].

Areas in the brain that are affected in cases of depression are the prefrontal cortex, cingulated gyrus, amygdala, hippocampus, thalamus, and hypothalamus. These brain regions are involved in the regulation of motivation, eating, sleeping, energy level, circadian rhythm, and responses to rewarding and aversive stimuli, which are all abnormal in depressed people [12].

Neurotransmitters that are depleted in patients with depression are serotonin that helps regulate sleep, appetite, and mood and inhibits pain. Reduced serotonin secretion is recorded in patients with depression [13]. Norepinephrine triggers anxiety and is involved in some types of depression. It determines motivation and reward. Norepinephrine and serotonin (5-HT) modulate subcortical and cortical functions that their shortage in states of depression and anxiety contributes to abnormalities in sleep, concentration, attention and memory, arousal states, appetite, and libido [14]. Furthermore, their modulation of the cortical-hippocampal-amygdala pathways regulates responses to aversive, stressful, and fearful experience along with modulation of the affective aspects of memory [15].

Several studies confirmed that major depression is associated with a state of reduced dopamine and treated with monoamine oxidase inhibitors [16]. Problems in dopamine secretion have been associated with psychosis, a severe form of distorted thinking, hallucinations, or delusions. Dopamine is thought to play a role during substance abuse [17].

Psychoactive substance use, such as khat (*Catha edulis*), alcohol, and tobacco have become the rising risk factors for major mental health problems worldwide [18]. Khat (*Catha edulis*), which is commonly used in the study area, contains a psychoactive substance, cathinone, which stimulates the central nervous system analogous to amphetamine. Its mechanism of action is believed to be mediated via its sympathetic like action in the body [19].

A common side effect of khat use is insomnia, a condition that the users sometimes try to overcome with intake of sedatives or alcohol. The withdrawal symptoms after prolonged khat use are lethargy, mild depression, slight trembling, and recurrent bad moods [20].

Although tobacco contains thousands of chemicals, the main active ingredient that acts in the brain and produces addiction is nicotine. Many of the effects of nicotine are produced through its action on both the central and peripheral nervous systems [21]. Alcohol, a brain depressant and intoxicant, is the most commonly used psychoactive substance by both mentally healthy people and mentally ill people [22].

Depression has severe consequences on the productive human force and social areas which calls the attention of care providers for early diagnosis, proper treatment, and intervention. Although the prevalence of khat use and its physiological and psychosocial effects were studied, there is still limited knowledge on the association between substance use and depression in Jimma town at community level. Therefore, the main aim of the present study is to determine the prevalence and severity of depression and its association with the risk factors such as substance use.

## 2. Research Methods

This cross-sectional, community based study was conducted from 15 February 2014 to 15 March 2014 in Jimma town. The town's total population was estimated to be 130,254. People of ≥18 years of age were included in the study.

Out of thirteen kebeles of Jimma town, six kebeles were selected using a lottery method and 650 households were selected using a systematic random sampling. Once the households were identified, one adult family member was selected randomly using the lottery method. The sample size was calculated using multistage sampling formula; the prevalence rate of depression (*p*) was taken to be 26.3% from the previous study [23], with the confidence level of 95% and margin of error of 5%. Contingency of 10% for the nonresponse rate was added and multiplied by 2 for the design effect assuming that the source population is heterogeneous and multistage sampling was taken.

Beck's Depression Inventory revision II (BDI-II) [24] screening questionnaire has been employed for screening depression and for grading its severity. BDI has a high degree of sensitivity and specificity for detecting depression. BDI scoring was conducted as follows: normal depression (0–13), mild depression (14–19), moderate depression (20–28), and severe depression (29–63). World Health Organization (WHO) drug addiction questionnaire [25] was adopted and modified to make it relevant to the objectives of the study. The magnitude of khat chewing, alcohol consumption, and cigarette smoking was evaluated using this questionnaire.

To improve the quality of the data, the questionnaire was pretested on 32 randomly selected subjects who were not included in the study samples. Questionnaires were translated from the English language into local language and back-translated to English language for the sake of consistency. A two-day training was given for 6 psychiatric nurses as data collectors. The questionnaires were checked for their completeness during data cleaning and the incomplete ones were discarded. Data analysis was carried out using SPSS Version 20.0 for Windows. Frequency tables were used to summarize categorical data. The associations between predictor and outcome variables were measured using Chi-square test. Multivariate logistic regression analysis was conducted to control confounders and to identify the independent contribution of each variable to the outcome variable.

Ethical clearance was obtained from Jimma University Ethical Review Board. Participants' consent was taken and confidentiality was maintained.

### 2.1. Operational Definitions

First, we define substances use: substances that are commonly used in the study site are alcohol, khat, cigarette, and shisha. Second, we define current chewers: they represent the proportion of respondents who were chewing khat within 30 days before the study. Third, we define nonchewers: they represent the proportion of respondents who were not chewing khat within 30 days before the study. Fourth, we define current smokers: They represent the proportion of respondents who were smoking cigarettes within 30 days before the study. Fifth, we define nonsmokers: they represent the proportion of respondents who were not smoking cigarettes within 30 days before the study. Sixth, we define current alcohol users: they represent the proportion of respondents who were drinking alcohol (beer, whisky, local araki, or gin) within 30 days before the study. Seventh, we define lifetime drinkers: they represent the proportion of respondents who had ever drunk alcohol in their lifetime. Eighth, we define non-alcohol users: they represent the proportion of respondents who were not drinking alcohol within 30 days before the study. Shisha is an oriental tobacco pipe with a long, flexible tube connected to a container, where the smoke is cooled by passing through water. Shisha smoking, also called hookah, water pipe, or hubble-bubble smoking, is a way of smoking tobacco, sometimes mixed with fruit or molasses sugar, through a bowl and hose or tube.

## 3. Results

**Figure 1:**
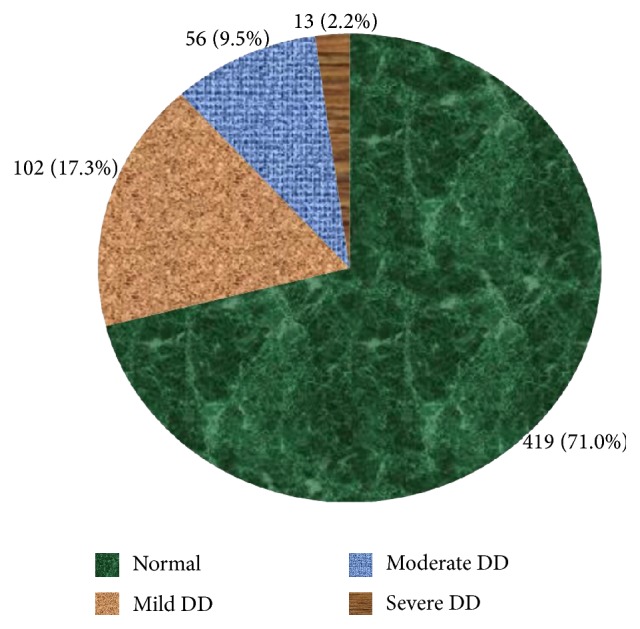
Severity of depression disorder in Jimma town, Southwest Ethiopia (*n* = 590). According to Beck's Depression Inventory, cutoff value ≤13 is normal; score of 14–19 is mild depression; score of 20–28 is moderate depression; score of 29–63 is severe depression. Four hundred nineteen participants were normal and 29% had depression, out of which 17.3% had mild depression, 9.5% had moderate depression, and 2.2% had severe depression.

Among 650 respondents, the participation rate was 590 (90.8%). The current prevalence of depression was 171 (29.0%). According to BDI-II grading scale, 102 (17.3%) participants had mild depression, 56 (9.5%) participants had moderate depression, and 13 (2.2%) participants had severe depression, as presented in Figure 1. The magnitude of depression increases with age (Table 1). The prevalence of depression increases to 56.4% as the age advances above 55 years. Age above 55 years is 5.9 times more likely to have depression than lower age groups.

As per gender, in comparison with males, significantly larger proportion, 90 (52.6%), of females had depression (AOR = 2.43; 95% CI: 1.43, 4.13). The highest prevalence of depression was recorded among widows (85.0%) and illiterates (64.4%) as presented in Tables 1 and 3.

Out of the total respondents, 200 (33.9%) khat chewers, 203 (34.4%) alcohol users, 60 (10.2%) cigarette smokers, and 22 (3.7%) shisha users were recorded. One hundred four (52.0%) khat chewers, 38 (63.3%) cigarette smokers, 75 (36.9%) alcohol users, and 15 (68.2%) shisha smokers had depression episodes. There was a significant association between depression and substance use (*p* < 0.05) as shown in Tables 2 and 3.

Those sociodemographic and substance use related variables that have shown a significant association with depression episodes were tested by multivariate logistic regression analysis. Age, sex, marital status, educational status, khat chewing, cigarette smoking, and shisha usage were found to be the risk factors for depression as presented in Table 3. The logistic regression model revealed that the odds of developing depression for 55 years of age and above was as follows: AOR = 5.94; 95% CI: 2.26–15.58. Regarding marital status, widowed individuals were five times more likely to develop depression in comparison with the singles (AOR = 5.18; 95% CI: 1.18–22.76). Illiterate individuals were nine times more likely to develop depression as compared to educated people (AOR = 9.06; 95% CI: 2.96–27.75).

Similarly, khat chewers had tenfold risk of developing depression as compared to nonchewers (AOR = 10.07; 95% CI: 5.57–18.25). Cigarette smokers were approximately three times more likely to develop depression compared to nonsmokers (AOR = 3.15; 95% CI: 1.51–6.58) and shisha users had threefold higher risk for having depression compared to non-shisha users (AOR = 3.04; 95% CI: 1.01–9.19).

## 4. Discussion

Burden of depression poses a substantial public health challenge. In the present study, the current prevalence of depression in the community was 29%. This result is in agreement with the study done in Uganda which has reported prevalence of depression of 29.3% [26]. A slightly lower prevalence of depression compared to the current finding has been reported by studies conducted in Ethiopia and France: 26.3% [27] and 26.5% [28], respectively. A much lower prevalence of depression was reported in studies conducted in South Africa and Malaysia: 9.7% [29] and 6.3% [30], respectively. A much higher prevalence of depression, 54.5%, was reported in a study conducted in Nigeria [31]. The possible explanation for the higher prevalence of depression disorder in the present study could be associated with the high production distribution and consumption of khat. Khat is grown abundantly in Jimma area. Many people chew khat in this place for different purposes. In most cases, khat chewing is accompanied by coffee drinking, cigarette smoking, and shisha usage and after these substances people used to drink alcohol to reduce the excitatory effect of khat and nicotine. Regular usage of all these substances could be the potential predisposing factors for depression.

In the current study, advanced age is strongly associated with the occurrence of depression and the result of this study is consistent with other previous findings conducted in USA [32] and Malaysia [30]. Age of 55 years and above was approximately six times more likely to develop depression as compared with age of 18–24 years. This could be due to disturbances of sleep, appetite, and sexual activities and due to an imbalance of neurotransmitters in the brain for nerve impulse transmission as age increases. Depressive symptoms are significantly higher among elders above the age of 55 years. The possible explanation is that as the age advances, several problems related to aging come into picture including deterioration of body functions and hormonal secretions and metabolic activities, decreased quality of life, and higher mortality from comorbid medical conditions which contribute to the development of depression.

In the present study, females were found to be twice more vulnerable to depression than males. This report is consistent with a study conducted in Turkey [33]. This could be due to a low social and economic status, cultural discrimination, the affective nature of females response to stressful life, and hormonal changes during puberty; postpartum and postmenopausal periods and taking contraceptive pills have been incriminated as possible factors for the higher prevalence of depression among women.

According to the present findings, depression was significantly associated with educational status. The prevalence of depression was nine times higher among illiterates compared to educated samples. The result is in agreement with studies conducted in South Africa [29] and Turkey [33]. The possible explanation for this could be the fact that individuals with low socioeconomic and educational status were given less value to their self-esteem and live a stressful life as compared with educated individuals. In addition, educated people have better understanding of the risk factors of depression compared to illiterates.

In this study, depression has shown a significant association with marital status. Widowed individuals were five times more likely to develop depression as compared to single individuals. The result of this report was in line with the study conducted in Ethiopia [27, 34, 35]. This could be due to the fact that the loss of a spouse or lovers has been identified as one of the most stressful life events, requiring more psychological therapy compared to many others.

Depression was significantly associated with khat chewing. The probability of developing depression episodes among khat chewers is tenfold higher than that among nonchewers. This result is consistent with the study conducted in Jimma University [18, 23] and with WHO expert analysis [36]. Some authors propose that depression episodes disappeared during khat chewing sessions [22]. But these depressive symptoms crop up on cessation of khat use. This might be due to the fact that khat contains psychoactive chemicals cathinone and cathine that have amphetamine like action in the brain which activates the release of monoaminergic neurotransmitters such as dopamine [37] in the limbic system, resulting in reward sensations but after the cessation of khat, it leads to depression.

The result of the present study indicated that depression shows a significant association with current cigarette smoker. This result demonstrates that smokers have developed depression symptom three times greater than nonsmokers. The present finding is in agreement with the study conducted by Pasco et al. [38]. However, other works in literature explained that depression and smoking show bidirectional relationship [39]. Substance use increases the risk of major depressive disorder [40]. Persons with major depression tend to abuse substances and have difficulties when they try to stop. There are thousands of chemicals other than nicotine present in cigarette smoke, of which one or several may affect mood in the same way as a group of antidepressant medications called monoamine oxidase inhibitors or (MAOIs) does [41]. These MAOIs effectively increase levels of specific neurotransmitters involved in the regulation of mood. Smoking, therefore, may be one way for depressed individuals to alleviate depressive symptoms.

## 5. Conclusion

To sum up, the magnitude of depression episodes recorded so far was found to be a moderate public health problem. The strongest association was recorded in the present study between depressive episodes and substance use. Chronic regular use of a psychoactive plant, khat, could be a potential risk factor. Khat chewing is accompanied by cigarette smoking and alcohol drinking. Avoidance of risk factors for depression is such a commendable preventive measure. Psychological and pharmacological therapies are equally important as well.

## Figures and Tables

**Table 1:** Association between depression and sociodemographic variables in Jimma town, Southwest Ethiopia (*n* = 590).

Variables		Depression			*χ* ^2^	*p* value
		Total, *n* = 590 *N* (%)	Yes, *n* = 171 *n* (%)	No, *n* = 419 *n* (%)		
Sex	Male	290 (49.1)	81 (27.9)	209 (72.1)		
	Female	300 (50.9)	90 (30.0)	210 (70.0)	31.00	0.001

Age	18–24	183 (63.1)	33 (18.0)	150 (82.0)		
	25–34	188 (31.9)	48 (24.5)	140 (74.5)		
	35–44	102 (17.3)	33 (32.4)	69 (67.6)	36.46	0.000
	45–54	62 (10.5)	26 (41.9)	36 (58.1)		
	≥55	55 (9.3)	31 (56.4)	24 (43.6)		

Marital status	Single	242 (41.0)	61 (25.2)	181 (74.8)		
	Married	312 (52.9)	84 (23.9)	228 (73.1)	38.59	0.000
	Divorced	16 (2.7)	9 (56.2)	7 (43.8)		
	Widowed	20 (3.4)	17 (85.0)	3 (15.0)		

Occupation	Merchant	213 (36.1)	51 (23.9)	162 (76.1)		
	Employed	61 (10.3)	21 (34.4)	40 (65.6)		
	Unemployed	56 (9.5)	18 (32.1)	38 (67.9)	36.06	0.000
	Housewife	134 (22.7)	55 (41.0)	79 (59.0)		
	Students	102 (17.3)	15 (13.8)	94 (86.2)		
	Others^*∗*^	24 (4.1)	11 (64.7)	6 (35.3)		

Educational status	Illiterate	45 (7.6)	29 (64.4)	16 (35.6)		
	1° School	140 (23.7)	49 (35.0)	91 (65.0)		
	2° School	207 (35.1)	50 (27.1)	151 (72.9)	29.47	0.000
	Certificate	38 (6.4)	11 (28.9)	27 (71.1)		
	Diploma	84 (14.2)	12 (14.3)	72 (85.7)		
	Degree plus	76 (12.9)	14 (18.4)	62 (81.6)		

^*∗*^Farmers, pension.

**Table 2:** Association between depression and substance use in Jimma town, Southwest Ethiopia (*n* = 590).

Variables		Depression			*χ* ^2^	*p* value
		Total, *n* = 590 *N* (%)	Yes, *n* = 171 *n* (%)	No, *n* = 419 *n* (%)		
Current khat chewing	Yes	200 (33.9)	104 (52.0)	96 (48.0)	77.88	0.000
	No	390 (66.1)	67 (17.2)	323 (82.8)		

Current cigarette smoking	Yes	60 (10.2)	38 (63.3)	22 (36.7)	38.29	0.000
	No	530 (89.8)	133 (25.1)	397 (74.9)		

Current alcohol intake	Yes	203 (34.4)	75 (36.9)	128 (63.1)	9.53	0.002
	No	387 (65.6)	96 (24.8)	291 (75.2)		

Current shisha use	Yes	22 (3.7)	15 (68.2)	7 (31.8)	17.06	0.000
	No	568 (96.3)	156 (27.5)	412 (72.5)		

**Table 3:** Multivariate logistic regression analysis for the association of different variables with depression episode in Jimma town, Southwest Ethiopia (*n* = 590).

Variables	Categories	Total respondents *n* (%)	Depression episodes *n* (%)	AOR (95% CI)	*p* values
Age	18–24	183 (31.0)	131 (31.0)	1.00	
	25–34	188 (31.9)	188 (31.9)	0.89 (0.46–1.73)	0.732
	35–44	102 (17.3)	102 (17.3)	1.55 (0.70–3.41)	0.281
	45–54	62 (10.5)	62 (10.5)	3.04 (1.24–7.41)	0.015
	55+	55 (9.3)	55 (9.3)	5.94 (2.26–15.58)	0.000

Sex	Male	290 (49.2)	290 (49.2)	1.00	
	Female	300 (50.9)	300 (50.8)	2.43 (1.43–4.13)	0.001

Marital status	Single	242 (41.0)	242 (41.0)	1.00	
	Married	312 (52.9)	312 (52.9)	0.75 (0.42–1.35)	0.335
	Divorced	16 (2.7)	16 (2.7)	1.49 (0.41–5.48)	0.547
	Widowed	20 (3.4)	20 (3.4)	5.18 (1.18–22.77)	0.029

Education	Illiterate	45 (7.6)	45 (7.6)	9.06 (2.96–27.75)	0.000
	Primary	140 (23.7)	140 (23.7)	4.13 (1.70–10.08)	0.002
	Secondary	207 (35.1)	207 (35.1)	3.14 (1.38–7.12)	0.006
	Certificate	38 (6.4)	38 (6.4)	4.36 (1.41–13.43)	0.010
	Diploma	84 (14.2)	84 (14.2)	1.00 (0.37–2.74)	1.000
	Degree+	76 (12.9)	76 (12.9)	1.00	

Current khat chewing	Yes	200 (33.9)	104 (52%)	10.07 (5.57–18.25)	0.000
	No	390 (66.1)	67 (17.2%)	1.00	

Current cigarette smoking	Yes	60 (10.2)	38 (63.3%)	3.15 (1.51–6.58)	0.002
	No	530 (89.8)	133 (25.1%)	1.00	

Current alcohol intake	Yes	203 (34.4)	75 (36.9%)	1.05 (0.63–1.76)	0.856
	No	387 (65.6)	96 (24.8%)	1.00	

Current shisha use	Yes	22 (3.7)	15 (68.2%)	3.04 (1.01–9.19)	0.049
	No	568 (96.3)	156 (27.5%)	1.00	

1.00 = reference categories. All variables were adjusted to differentiate between predictors and confounders.
